# Commentary: Fungal lifestyle reflected in serine protease repertoire

**DOI:** 10.3389/fmicb.2018.00467

**Published:** 2018-03-13

**Authors:** Ronivaldo R. da Silva

**Affiliations:** Faculdade de Ciências Farmacêuticas de Ribeirão Preto, Universidade de São Paulo, Ribeirão Preto, Brazil

**Keywords:** biotechnology, catalysis, fungal enzyme, lifestyle, protease

Biochemical diversity and adaptability to different environments are well-known biological characteristics of filamentous fungi. Thus, these microorganisms are widely distributed throughout the biosphere, integrating saprophytic (essential for cycling of nutrients), pathogenic, and symbiotic (with various animals and plants) lifestyles into the environment.

Their biological versatility is aligned with their ability to secrete a wide array of enzymes that degrade macromolecules available in the growing environment (da Silva et al., 2014). From the perspective of the secreted enzyme repertoire, a recent noteworthy article titled “Fungal lifestyle reflected in serine protease repertoire” (Muszewska et al., 2017) exhibited a phylogenetic distribution of serine peptidases represented in the fungal kingdom. The paper provides a comprehensive analysis of the presence of the different serine peptidases families in a large number of fungal species. Based on statistical analyses, the authors also draw associations between fungal lifestyle and serine peptidase repertoire.

The article shows the evolutionary trajectory of serine peptidases. However, despite what is proposed in the paper, the association between the fungal lifestyle and the serine peptidase repertoire has not yet been well-discussed, especially with respect to the biochemical versatility of these enzymes, which makes them fundamental for broad spectrum action over different proteins in the growth medium and play an essential role in the intracellular processing of proteins and peptides. Thus, the aim of this commentary is to add some further aspects about the catalytic properties of serine peptidases and their representative importance for proteolysis in the fungal growth environment. This characteristic is closely associated with exploration and adaptation to the habitat, which has been demonstrated by the ability of fungi to take advantage of the protein sources present in the environment, eventually contributing to their ecological success. Therefore, an enzyme with a broad spectrum of action over different proteins, and under different pH and temperature conditions stands out as a fundamental tool for the survival of the species (Di Cera, 2009; da Silva, 2017).

Based on several studies conducted over the years, serine peptidases represent the best-known group of proteolytic enzymes among the seven catalytic types of this group of enzymes that are crucial for all organisms (Di Cera, 2009; da Silva, 2017). Metallopeptidase, cysteine peptidase, and aspartic peptidase are also enzymes that are studied in depth. What makes serine peptidases crucial tools for different fungal lifestyles from an enzyme catalysis standpoint?

To answer this question, the enzyme catalytic properties needs to be detailed. This catalytic type of proteolytic enzyme is characterized by the conventional catalytic triad, His, Ser, and Asp, where the serine residue is responsible for the nucleophilic cleavage of the peptide bond (Di Cera, 2009). Some serine peptidases have modifications in the catalytic triad or dyad. However, serine residues are characteristic in the active sites and they define the mechanism of action of these enzymes (Hedstrom, 2002; da Silva, 2017).

Based on the different fungal lifestyle, it is important to highlight the versatility of serine peptidases in the degradation of proteins present in fungal growth media. When observing pathogenic fungi in plants and animals, serine peptidases are important to hydrolyze tissue proteins favoring fungal metabolism and the spread of infection.

In saprophytic fungi, serine peptidases are also extremely important in decomposing biomass. Thus, serine peptidases in fungi isolated from the soil have been frequently characterized. Numerous investigations have described this in the literature. The same is also observed in symbiotic fungi associated with animals and plants. Notably, it is important to mention that symbiotic fungi have less subtilisins and other serine peptidase family members than saprotrophic and pathogenic fungi (Zanphorlin et al., 2011; da Silva et al., 2013a,b, 2014; Graminho et al., 2013; Biaggio et al., 2016; Ida et al., 2016; da Silva, 2017).

The catalytic properties of serine peptidases deserve more attention. Many investigations have described the effects of the broad pH and temperature range on the enzymatic activity of fungal serine peptidases (da Silva et al., 2013a,b, 2014; Graminho et al., 2013; Biaggio et al., 2016; Ida et al., 2016; da Silva, 2017), which can be categorized as endo or exopeptidases (da Silva, 2017). It is possible to observe reports of fungal serine peptidases with optimum activity varying between pH 6.0–11.0 and temperatures between 50 and 70°C. In general, extracellular serine peptidases secreted by fungi have a broad spectrum of action over different proteins. Some studies have demonstrated the secretion of serine peptidases acting on different amino acid sequences (Graminho et al., 2013; da Silva et al., 2014).

Some studies have indicated the broad spectrum of protein hydrolysis by serine peptidases on different peptide substrate sequences. This was observed by Zanphorlin et al. (2011) in *Myceliophthora* spp., Graminho et al. (2013) in *Penicillium waksmanii*, and Watson et al. (2011) and da Silva et al. (2014) in *Aspergillus fumigatus*. In another study regarding substrate specificity, Biaggio et al. (2016) reported that an extracellular serine peptidase from *Aspergillus terreus* exhibited low specificity at the S_1_ subsite, accepting several amino acids in this position and having the highest catalytic efficiency (*k*_cat_/*K*_M_) when threonine and tyrosine were located at P_1_.

This demonstrates the importance of these enzymes in the degradation of proteins present in the fungal growth substrate, the proteolysis of which favors the assimilation of amino acids fundamental for the survival of the microorganism. Here, it is important to emphasize that the biochemical characteristics highlighting the performance of these enzymes correlate with optimal growth conditions for most fungi, thereby establishing their importance with respect to fungal ubiquity in the biosphere.

It is also essential to point out that the broad pH and temperature range for enzymatic activity, and the versatility in hydrolyzing a broad spectrum of proteins, establish extracellular serine peptidases as essential components in the exploration of nutritional environmental resources, which facilitate the fungal kingdom with representative species with varying lifestyles.

It is extremely important to highlight the scientific contribution of the paper authored by Muszewska et al. (2017). Here, an argument was made to better relate the catalytic versatility of serine peptidases as fundamental tools for different fungal lifestyles and their importance to the exploration of different habitats on the planet.

## Author contributions

The author confirms being the sole contributor of this work and approved it for publication.

### Conflict of interest statement

The author declares that the research was conducted in the absence of any commercial or financial relationships that could be construed as a potential conflict of interest.

## References

[B1] Biaggio R. T. Silva R. R. Rosa N. G. Leite R. S. Arantes E. C. Cabral T. P.. (2016). Purification and biochemical characterization of an extracellular serine peptidase from *Aspergillus terreus*. Prep. Biochem. Biotechnol. 46, 298–304. 10.1080/10826068.2015.1031387 25830777

[B2] da Silva R. R. (2017). Bacterial and fungal proteolytic enzymes: production, catalysis and potential applications. Appl. Biochem. Biotechnol. 183, 1–19. 10.1007/s12010-017-2427-2 28160134

[B3] da Silva R. R. Ângelo T. Cabral H. (2013a). Comparative evaluation of peptidases produced by *Penicillium corylophilum* and *Penicillium waksmanii* in solid state fermentation using agro-industrial residues. J. Agric. Sci. Technol. B 3, 230–237.

[B4] da Silva R. R. de Freitas Cabral T. P. Rodrigues A. Cabral H. (2013b). Production and partial characterization of serine and metallo peptidases secreted by *Aspergillus fumigatus* Fresenius in submerged and solid state fermentation. Braz. J. Microbiol. 44, 235–243. 10.1590/S1517-83822013000100034 24159310 PMC3804204

[B5] da Silva R. R. Caetano R. C. Okamoto D. N. de Oliveira L. C. Bertolin T. C. Juliano M. A.. (2014). The identification and biochemical properties of the catalytic specificity of a serine peptidase secreted by *Aspergillus fumigatus* Fresenius. Prot. Pept. Lett. 21, 663–671. 10.2174/0929866521666140408114646 24725188

[B6] Di Cera E. (2009). Serine proteases. IUBMB Life 61, 510–515. 10.1002/iub.186 19180666 PMC2675663

[B7] Graminho E. R. da Silva R. R. de Freitas Cabral T. P. Arantes E. C. da Rosa N. G. Juliano L.. (2013). Purification, characterization, and specificity determination of a new serine protease secreted by *Penicillium waksmanii*. Appl. Biochem. Biotechnol. 169, 201–214. 10.1007/s12010-012-9974-3 23179282

[B8] Hedstrom L. (2002). Serine protease mechanism and specificity. Chem. Rev. 102, 4501–4523. 10.1021/cr000033x 12475199

[B9] Ida É. L. da Silva R. R. de Oliveira T. B. Souto T. B. Leite J. A. Rodrigues A.. (2016). Biochemical properties and evaluation of washing performance in commercial detergent compatibility of two collagenolytic serine peptidases secreted by *Aspergillus fischeri* and *Penicillium citrinum*. Prep. Biochem. Biotechnol. 47, 282–290. 10.1080/10826068.2016.1224247 27552601

[B10] Muszewska A. Stepniewska-Dziubinska M. M. Steczkiewicz K. Pawlowska J. Dziedzic A. Ginalski K. (2017). Fungal lifestyle reflected in serine protease repertoire. Sci. Rep. 7:9147. 10.1038/s41598-017-09644-w 28831173 PMC5567314

[B11] Watson D. S. Feng X. Askew D. S. Jambunathan K. Kodukula K. Galande A. K. (2011). Substrate specifity profiling on the *Aspergillus fumigatus* proteolytic secretome reveals consensus motifs with predominance of Ile/Leu and Phe/Tyr. PLoS ONE 6, 1–13. 10.1371/journal.pone.0021001 21695046 PMC3117871

[B12] Zanphorlin L. M. Cabral H. Arantes E. Assis D. Juliano L. Juliano M. A. (2011). Purification and characterization of a new alkaline serine protease from the thermophilic fungus *Myceliophthora* sp. Process Biochem. 46, 2137–2143. 10.1016/j.procbio.2011.08.014

